# Influence of irradiated dentin, biofilm and different artificial saliva formulations on root dentin demineralization

**DOI:** 10.1016/j.heliyon.2024.e36334

**Published:** 2024-08-14

**Authors:** Beatriz Martines de Souza, Aline Silva Braga, Mariele Vertuan, Susan Sassaki, Tamara Teodoro Araújo, Paulo Sergio da Silva Santos, Marilia Afonso Rabelo Buzalaf, Ana Carolina Magalhães

**Affiliations:** a Department of Biological Sciences, Bauru School of Dentistry, University of São Paulo, Bauru, São Paulo, Brazil; b Department of Surgery, Stomatology, Pathology and Radiology, Bauru School of Dentistry, University of São Paulo, Bauru, São Paulo, Brazil

**Keywords:** Biofilms, Dental caries, Head and neck neoplasms, Radiotherapy

## Abstract

The aim of this study was evaluated the influence of radiation as well as of new formulations of artificial saliva on the development of root dentin lesions. Bovine root samples were divided into: irradiated (70 Gy) dentin or not; the type of biofilm (from irradiated patient-experimental or non-irradiated patient-control) and the type of artificial saliva (for the condition irradiated dentin/biofilm from irradiated patient): Control Artificial Saliva (inorganic); Control Saliva + 1 mg/ml hemoglobin; Control Saliva +0.1 mg/ml cystatin; Control Saliva + hemoglobin + cystatin; Bioextra (positive control) and deionized water (DiW, negative control) (n = 12/group). Biofilm was produced using human biofilm and McBain saliva (0.2 % of sucrose, 37^o^ C and 5 % CO_2_); the treatments were done 1x/day, for 5 days. Colony-forming units (CFU) counting was performed; demineralization was quantified by transversal microradiography. Two-way ANOVA/Bonferroni or Sidak test for the comparison between biofilm x dentin and ANOVA/Tukey or Kruskal-Wallis/Dunn for comparing artificial saliva were done (p < 0.05). The type of biofilm had no influence on CFU and demineralization. Sound dentin under control biofilm presented the lowest Lactobacillus ssp. and Streptococcus mutans CFU and the lowest mean mineral loss (R) (25.6 ± 2.2; 23.7 ± 2.9 %) compared to irradiated dentin (26.1 ± 2.8; 28.1 ± 3.3, p < 0.004) for both types of biofilms (experimental and control, respectively). Bioextra was the only artificial saliva that reduced R (10.8 ± 2.5 %) and Lesion Depth (LD) (35 ± 15 μm) compared to DiW (17.3 ± 3.3 %, 81 ± 18 μm, p < 0.0001). Irradiation has impact on caries development; the experimental saliva were unable to reduce its occurrence.

## Highlights

• Irradiated dentin presents higher susceptibility to demineralization.
• Commercial Bioxtra® saliva is able to reduce radiation-related dentin caries.
• Hemoglobin and Cane CPI-5 has no antimicrobial/anticariogenic in this study model.

## 1 Introduction

Despite the technological advances related to radiotherapy (conformational and modulated) to minimize secondary radiation to tissues adjacent to the tumor, this side effect still occurs even at a lower intensity [1]. Considering that radiotherapy is the main treatment of the head and neck cancer (HNC), the side-effects may involve the oral tissues, such as salivary glands, oral mucosa and dental structures [2] with a negative impact on the health and quality of life of this population.

Dentin has shown significant morphological changes after radiotherapy [3], which may compromise adhesive restorations performed after the cancer treatment period [4]. There seems to be an increase in the expression and activity of MMP-2 and MMP-9 in irradiated dentin, suggesting greater susceptibility to degradation [5]. In addition, it is expected reduced salivary flow, increased viscosity, reduced buffering capacity and changes in concentrations of electrolytes and proteins with antimicrobial function in saliva. All these alterations can increase the individual susceptible to the development of dental caries [6].

The caries lesions incidence in patients submitted to head and neck radiotherapy is about 16 % higher after the first year, reaching up to 74 % higher after 7 years of treatment [7,8]. Interventions to prevent this type of dental disease are needed and very important to provide an improved quality of life to the patients.

Besides the strategies to control dental caries, such as sugar consumption control and oral hygiene with fluoride toothpaste, the use of artificial saliva has been an important strategy applied to control the symptoms of hyposalivation in these patients, especially with respect to oral dryness.

New studies have emerged regarding the identification of saliva proteins that form the acquired pellicle and could have acid-resistant action, with potential to be incorporated into dental products, such as artificial saliva [[9], [10], [11], [12]]. Cystatin, a typical salivary protein, and hemoglobin, a typical blood protein, were identified in saliva proteomics, showing to be acid-resistant and, consequently, to have a significant anti-caries role [9,10,13]. Sugarcane cystatin, known as Cane CPI-5, has been used as a substitute for human cystatin, due to its low cost, a good affinity for hydroxyapatite, high acid resistance [14] and antibiofilm effect *in vitro* [12]. Hemoglobin also has a strong affinity to hydroxyapatite [15] and good results against the initial erosion *in vitro* as with Cane CPI-5 [11].

The use of artificial saliva has been the primary strategy employed by dentists to manage symptoms of hyposalivation in these patients, particularly concerning dry mouth. Various types of artificial saliva or saliva substitutes are available in the market, some containing only minerals and humectants, while others comprise more complex formulas containing certain proteins [16,17]. However, it is observed that the vast majority of artificial saliva options lack the ability to control caries development [18,19], especially in root dentin, and in some cases, they have even an opposing effect [20]. In addition, no previous work has tested, either individually or in combination, the effect of irradiated teeth and biofilm from patients with radiation-induced xerostomia on the development of root caries lesions.

Therefore, the aim of this study was to test the influence of radiation on the development of root dentin caries as well as of new formulations of artificial saliva containing Cane CPI-5 and hemoglobin on the development of root dentin lesions, with the objective of reducing the development of radiation caries, focusing on its possible use by patients who are undergoing radiotherapy treatment of the HNC.

The primary null hypothesis is that there is no difference in either the type of dentin (sound or irradiated) or the quality of the biofilm (from irradiated patient-experimental or non-irradiated patient-control) regarding the development of dentin lesions using an *in vitro* microcosm biofilm model. The secondary null hypothesis is that there is no difference between the different formulations of artificial saliva compared to the negative control regarding their antimicrobial and anticaries action.

## 2 Materials and methods

### 2.1 Biofilm collection

This study was previously approved by the local Ethics Committee (CAAE: 97497318.00000.5417). Written informed consent was obtained from all individual participants included in the study. Biofilm considered control was collected from two healthy patients (1 male: 60 years old with 26 teeth and 1female: 56 years old with 26 teeth, without cancer) and pooled. The inclusion criteria followed previously reported protocols [21]. The collection of irradiated biofilms was performed from two donors (1 male: 65 years old with 20 teeth and 1 female: 57 years old with 24 teeth) who received the total head and neck 3D radiotherapy (final dose: 70 Gy), 5 months previously to the study, following inclusion criteria previously determined and described by de Souza et al. [21]. The biofilm was collected from the cervical root regions of all teeth without active caries lesions, using a curette, and stored in Eppendorf tubes kept on ice [21]. The entire collection procedure was conducted at the Clinical Research Center of the Institution. After that, the biofilms were diluted in 0.9 % saline solution (proportion 2 mg: 1 ml), and then vortexed (30 s) and sonicated (5 % amplitude, 3 pulses of 10 s each). Thereafter, 1 ml aliquots (70 % biofilm solution and 30 % glycerol) were prepared and stored at −80 °C [21,22].

### 2.2 Tooth specimen preparation and treatment groups

This study was first approved by ethics committee on animal research (CEUA, Number: 004/2018). The bovine teeth were donated by food manufacturing industry (Frigol S.A, Lençóis Paulista, São Paulo, SP, Brazil). One hundred and twenty bovine root samples (4 mm × 4 mm) were prepared and minimum polished (5 s), to allow bacterial adhesion [21]. The samples were randomly distributed to the groups, considering roughness values means (measured by using a contact profilometer - Mahr Perthometer, Göttingen, Germany and the software MarSurf XCR-20 -Mahr Perthometer, Göttingen, Germany) as criteria (mean: 0.28 ± 0.02 μm). Then, two parts of each third of the surface were covered with red nail polish (Love-Risqué®, Guarulhos, SP, Brazil) to allow later appropriate analysis of tooth demineralization by transverse microradiography - TMR. Of these, 96 dentin samples were irradiated with a total dose of 70 Gy while 24 were not irradiated (sound dentin). Thereafter, the samples were sterilized by exposure to ethylene oxide [21]. Statistical sample size (n = 12) was calculated by using http://powerandsamplesize.com/Calculators, based on previous study using irradiated dentin and microcosm biofilm model [21], in which the sample power was 97 %.

Forty-eight dentin samples (n = 24 irradiated and n = 24 non-irradiated- sound) were submitted to two distinct microcosm biofilm formations (Experimental-Biofilm - biofilm from irradiated patientes or Control Biofilm - biofilm from healthy patients no irradiated), totalizing four groups (n = 12/group): (1) Irradiated dentin and Experimental-biofilm; (2) Irradiated dentin and control-biofilm; (3) Sound dentin and Experimental-biofilm; (4) Sound dentin and control-biofilm.

While seventy-two samples of irradiated dentin were submitted to microcosm biofilm formation using biofilm from irradiated patients and divided into 6 treatments with artificial saliva (n = 12/group):

(A) Control Artificial Saliva: pH 5.6 – NaHCO_3_ (0.219 %); K_2_HPO_4_ (0.127 %); CaCI_2_.2H_2_O (0.0654 %); MgCl_2_.6H_2_O (0.0125 %); KCl (0.082 %); Methylparaben (0.01 %); Propylparaben (0.01 %); Carboxymethylcellulose (0.8 %);
(B) Control Saliva +1 mg/mL hemoglobin (human hemoglobin, Sigma Aldrich, Saint Louis, MI, USA, H7379) (pH 5.4) [11];
(C) Control Saliva +0.1 mg/mL sugarcane cystatin (Cane CPI-5) (pH 5.5) [14];
(D) Control Saliva +1 mg/mL hemoglobin +0.1 mg/mL sugarcane cystatin (Cane CPI-5) (pH 5.5);
(E) BioXtra® (positive control) (active components: lysozyme; lactoferrin, lactoperoxidase; colostrum extrac. Other ingredients: water, Propylene Glycol, Xylitol, Sodium Monofluorophosphate (1500 ppm F^−^), Poloxamer 407, Hydroxyethylcellulose, Aroma, Aloe Barbadensis Leaf Juice, EDTA, Lactic Acid, Sodium Benzoate, Limonene, Linalool, CI42090. Lifestream Pharma, Seneffe, Belgium) (pH 6.3);
(F) Deionized Water (DiW) (negative control) (pH 7.1).

**Fig. 1:**
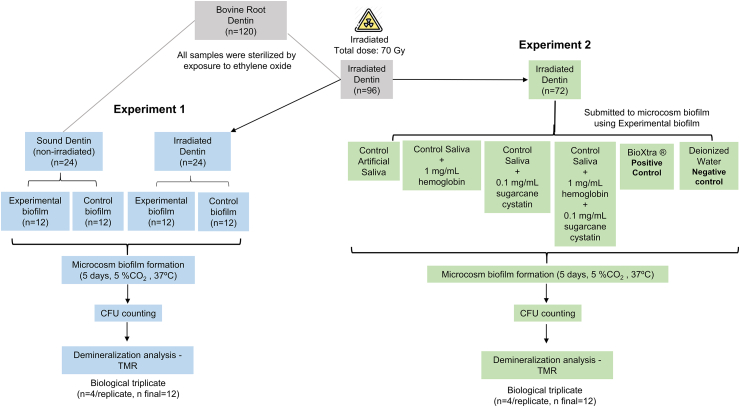
Flowchart of the study.

All the experiments were done in biological triplicate (n = 4/replicate, n final = 12). Fig. 1 shows a flowchart of the study with the division of the samples and experiments.

### 2.3 Microcosm biofilm formation

The biofilm-glycerol stock was diluted in McBain artificial saliva [23], at a ratio of 1:50 (inoculum) [21]. The microcosm biofilm was grown on the dentin samples placed into the 24-well plates, for five days. On the first day, each dentin sample was exposed to 1.5 ml of inoculum for 8 h. After this time, the inoculum was removed, the samples were washed with PBS (1.5 ml, 5 s) and received 1.5 ml fresh medium (McBain Saliva with 0.2 % of sucrose) until completing 16 h. From the second to the fifth day, the medium with sucrose was changed once a day and the plates were incubated at 5 % CO_2_ and 37 °C [12,21]. Treatments with artificial saliva were performed between medium changes, once/day for 1 min, during the following 4 days of microcosm biofilm formation (schematic diagram of biofilm microcosm in Online Resource 1).

### 2.4 Colony-forming unit (CFU) counting

After 5 days, the microbial suspension from each well plate, obtained by adding 1 ml of 0.89 % NaCl solution over the formed biofilm, was sonicated (Sonifier Cell Disruptor B-30, Branson) for 30 s at 20 W [24]. Bacterial suspensions were diluted (10^−4^ or 10^−5^) and spread on Petri dishes (25 μL/dish) and then, the dishes incubated under 5 % CO_2_ and 37 °C for 48 h [12,21]. The four agar culture media used were [21]: 1) brain heart infusion agar (BHI; Difco, Detroit, USA) for total microorganisms (dilution factor 10^−5^); (2) mitis salivarius agar (MSA; Neogen, Indaiatuba, Brazil) for total *streptococci (*dilution factor 10^−4^); (3) SB-20M for *mutans streptococci* (*S. mutans* and *S. sobrinus*) (dilution factor 10^−4^); and (4) MRS (Kasvi, Curitiba, Brazil) for *Lactobacillus* sp. (dilution factor 10^−5^). After 48h, the CFU numbers were counted and used, together with the dilution factor, to calculate the total CFU for each type of microorganism per group. The data were transformed in log_10_ CFU/mL [21].

### 2.5 Transverse microradiography (TMR) - demineralization analysis

Dentin samples were cleaned with sterile gauze, transversally sectioned and polished (100–120 μm thickness). The procedure of exposure to X-ray (20 kV and 20 mA, Softex, Tokyo, Japan), development and analysis of the microradiography plates was performed as previously described [21], using the TMR from Inspektor Research System (Amsterdam, Netherlands) (schematic diagram of the sample preparation for TMR in Online Resource 2). The mineral content was calculated assuming 50 vol% of mineral content for sound dentin and that the lesion depth ends when dentin contains around of 47.5 % of mineral volume [21]. The integrated mineral loss (ΔZ, %vol. μm), the average mineral loss over the lesion depth (mean mineral loss, R, %vol) and the lesion depth (LD, μm) were calculated [21].

### 2.6 Statistical analysis

The data were submitted to statistical analysis using the Graph Pad Prism 7.04 program (San Diego, USA). (p < 0.05). The normal distribution and homogeneity were checked using Kolmogorov–Smirnov and Bartlett tests, respectively. For the comparison between biofilm (from irradiated or healthy patient) x dentin (irradiated or sound) 2-way ANOVA/Bonferroni test (CFU counting) and 2-way ANOVA/Sidak test (TMR parameters) were done. For comparison between treatments with artificial saliva, ANOVA/Tukey test was applied for CFU counting (total microorganisms and *Lactobacillus* spp) and for TMR parameters (R and LD) and Kruskal-Wallis/Dunn for CFU counting (total streptococci and *mutans streptococci*) and for the Integrated Mineral Loss (ΔZ).

## 3 Results

### 3.1 CFU counting

#### 3.1.1 Experiment 1

There was no difference between the type of dentin and type of biofilm on the growth of total microorganisms. Regarding total streptococci, sound dentin had a higher number of CFU compared to irradiated dentin, in the case of those exposed to biofilm from irradiated patient, but no difference was seen in the case of biofilm from health patient. For *Lactobacillus* sp. and *mutans streptococci,* there was no difference between the type of dentin (irradiated or sound), while the biofilm from irradiated patient had a higher number of CFU compared to biofilm from health patient placed on sound dentin. For irradiated dentin, no difference was found in CFU counting (Table 1).

**Table 1:** Mean ± SD of CFU counting (log_10_ CFU/mL) of Total Microorganisms, *Lactobacillus* sp. (10^−5^), Total *streptococci* and *Streptococcus mutans/Streptococcus sobrinus* (10^−4^) of Microcosm Biofilm Produced from Different Sources of Biofilm on Distinct Dentin Substrates.

	total microorganisms	*Lactobacillus* sp.	total *streptococci*	*S. mutans/S. sobrinus*
**Irritated dentin and Experimental -biofilm**	6.66 ± 0.23^Aa^	7.15 ± 0.37 ^Aa^	6.82 ± 0.35 ^Aa^	6.91 ± 0.33 ^Aa^
**Irritated dentin and Control- biofilm**	6.43 ± 0.48 ^Aa^	6.86 ± 0.37 ^Aa^	6.81 ± 0.22 ^Aa^	6.88 ± 0.13 ^Aa^
**Sound dentin and Experimental -biofilm**	6.67 ± 0.37 ^Aa^	7.13 ± 0.38 ^Aa^	7.28 ± 0.35 ^Bb^	7.08 ± 0.30 ^Ab^
**Sound dentin and Control- biofilm**	6.44 ± 0.36 ^Aa^	6.72 ± 0.39 ^Ab^	6.95 ± 0.31 ^Aa^	6.72 ± 0.28 ^Aa^

Different uppercase letters in the same column indicate a significant difference between dentin types. Different lowercase letters in the same column indicate a significant difference between biofilm types. 2 way-ANOVA/Bonferroni (total microorganisms: interaction p = 0.988, biofilm type p = 0.037, dentin type p = 0.969; *Lactobacillus* spp.: interaction p = 0.573, biofilm type p = 0.003, dentin type p = 0.471; total *streptococci*: interaction p = 0.085, biofilm type p = 0.060, dentin type p = 0.002; *mutans streptococci*: interaction p = 0.045, biofilm type p = 0.020, dentin type p = 0.950).

#### 3.1.2 Experiment 2

With respect to the artificial saliva formulations comparisons, for total *streptococci* and *mutans streptococci*, treatments with experimental saliva, especially those containing hemoglobin, presented a higher CFU counting than the negative control (DiW), differing significantly (Table 2). The positive control (BioXtra®) significantly reduced the CFU counting for all analyzed species compared to the experimental saliva; however, it did not differ from negative control (DiW) with respect to *Lactobacillus* sp., *mutans streptococci* and total *streptococci* growth.

**Table 2:** Mean ± SD of CFU Counting (log_10_ CFU/mL) of Total Microorganisms and *Lactobacillus* sp. (10^−5^) and Median (interquartile range) of Total *streptococci* and *Streptococcus mutans/Streptococcus sobrinus* (10^−4^) of Microcosm Biofilm from irradiated patients treated with Different Saliva Formulations.

	Total microorganisms	*Lactobacillus* sp.	total *streptococci*	*S. mutans/S. sobrinus*
**Control Artificial Saliva**	6.69 ± 0.22^B^	7.20 ± 0.32^B^	7.54 (0.34)^BC^	7.30 (0.25)^C^
**Control Saliva + 1 mg/mL hemoglobin**	6.69 ± 0.32^B^	7.29 ± 0.22^B^	7.65 (0.16)^C^	7.42 (0.34)^C^
**Control Saliva + 0.1 mg/mL cystatin**	6.80 ± 0.32^B^	7.30 ± 0.33^B^	7.61 (0.46)^BC^	7.28 (0.30)^C^
**Control Saliva + 1 mg/mL hemoglobin + 0.1 mg/mL cystatin**	6.64 ± 0.38^B^	7.22 ± 0.37^B^	7.53 (0.19)^BC^	7.11 (0.37)^BC^
**BioXtra® (positive control)**	6.21 ± 0.23^A^	6.83 ± 0.19^A^	6.88 (0.56)^A^	6.63 (0.64)^A^
**Deionized Water**	6.61 ± 0.31^B^	7.11 ± 0.28^AB^	7.22 (0.59)^AB^	6.75 (0.54)^AB^

Different letters show statistical difference between treatments. ANOVA/Tukey: total microorganisms (p = 0.0003); *Lactobacillus* spp. (p = 0.0022). Kruskal-Wallis/Dunn: total *streptococci* (p < 0.0001); *mutans streptococci* (p < 0.0001).

### 3.2 TMR analysis

#### 3.2.1 Experiment 1

**Fig. 2:**
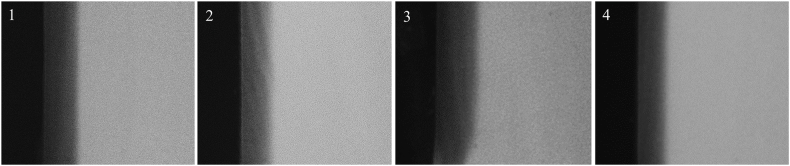
Representative TMR images of dentin sample from each of the following groups:(1) Irritated dentin and experimental-biofilm; (2) Irritated dentin and Control-biofilm; (3) Sound dentin and experimental-biofilm; (4) Sound dentin and Control-biofilm.

There were no statistically significant differences found between the type of biofilm and the type of dentin regarding the integrated mineral loss (ΔZ) and lesion depth (LD). When analyzing the mean mineral loss (R), sound dentin exposed to any type of biofilm showed less demineralization compared to irradiated dentin for both biofilm types (experimental and control) (Table 3). In Fig. 2, it is possible to observe the formation of a more radiopaque pseudo-intact superficial layer for all groups over a demineralized sub-surface area (more radiolucent area), characterizing the lesions as subsurface ones.

**Table 3:** Mean ± SD of the Integrated Mineral Loss (ΔZ, vol%. μm), Lesion Depth (LD, μm) and the Mean Mineral Loss (R, vol%) of Irradiated and Sound Dentin Submitted to Demineralization from Different Biofilm Sources.

	ΔZ (vol%.μm)	LD (μm)	R (vol%)
**Irritated dentin and Experimental-biofilm**	3186 ± 676	123 ± 27	26.1 ± 2.8^Aa^
**Irritated dentin and Control- biofilm**	3494 ± 516	126 ± 25	28.1 ± 3.3^Aa^
**Sound dentin and Experimental-biofilm**	3174 ± 914	114 ± 26	25.6 ± 2.2^Aa^
**Sound dentin and Control- biofilm**	3302 ± 601	134 ± 27	23.7 ± 2.9^Ba^

Different uppercase letters in the same column indicate a significant difference between dentin types. Different lowercase letters in the same column indicate a significant difference between biofilm types. Absence of letters shows no statistical difference.

2 way-ANOVA/Tukey (ΔZ: interaction p = 0.655; biofilm type p = 0.281; dentin type p = 0.613; LD: interaction p = 0.275; biofilm type p = 0.131; dentin type p = 0.990; R: interaction p = 0.021; biofilm type p = 0.960; dentin type p = 0.0042).

#### 3.2.2 Experiment 2

**Fig. 3:**
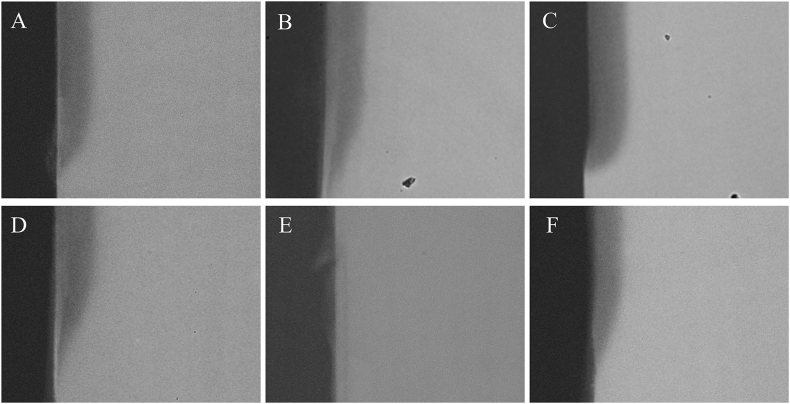
Representative TMR images of irradiated dentin sample with irradiated biofilm from each of the following treatments: (A) Control Artificial Saliva; (B) Control Saliva +1 mg/mL hemoglobin; (C) Control Saliva +0.1 mg/mL sugarcane cystatin; (D) Control Saliva +1 mg/mL hemoglobin +0.1 mg/mL sugarcane cystatin; (E) BioXtra® (positive control); (F) Deionized Water (negative control).

The experimental saliva formulations were not able to reduce the dentin lesion development under this experimental model. Only the positive control (BioXtra®) significantly reduced ΔZ, LD and R compared to the experimental salivas and the DiW (Table 4). In the representative images of Fig. 3, E image (BioXtra) shows the smallest radiolucent area (demineralized area) compared to the others.

**Table 4:** Median (interquartile range) of the Integrated Mineral Loss (ΔZ, vol%. μm) and Mean ± SD of the Lesion Depth (LD, μm) and the Mean Mineral Loss (R, vol%) of Irradiated Dentin Submitted to Microcosm Biofilm from irradiated patients treated with Different Saliva Formulations.

	ΔZ (vol%.μm)	LD (μm)	R (vol%)
**Control Artificial Saliva**	2390 (485)^C^	120 ± 17^C^	20.2 ± 4.2^B^
**Control Saliva + 1 mg/mL hemoglobin**	2240 (358)^BC^	113 ± 20^C^	19.4 ± 2.1^B^
**Control Saliva + 0.1 mg/mL cystatin**	2025 (1258)^BC^	112 ± 23^C^	19.9 ± 4.4^B^
**Control Saliva + 1 mg/mL hemoglobin + 0.1 mg/mL cystatin**	2395 (1408)^BC^	131 ± 30^C^	21.1 ± 4.4^B^
**BioXtra® (positive control)**	445 (420)^A^	35 ± 15^A^	10.8 ± 2.5^A^
**Deionized Water**	1520 (390)^AB^	81 ± 18^B^	17.3 ± 3.3^B^

Different letters show statistical difference between treatments. Kruskal-Wallis/Dunn: ΔZ (p < 0.0001). ANOVA/Tukey: LD (p < 0.0001); R (p < 0.0001).

## 4 Discussion

Irradiated biofilm has no influence on dentin lesion formation while irradiation of dentin increases the susceptibility to demineralization under the tested model. Therefore, the primary null hypothesis was partially rejected. There are changes in the composition and structure of root dentin at exposure to doses greater than 60 Gy with reduction of the mineral/organic matrix ratio [25], in addition, to possible changes in the composition of the organic and collagen content [[25], [26], [27]]. The mineral content also appears to be altered due to the radiation, with a decrease in the calcium and phosphate ratio, changing dentin hardness value [3]. The cited alterations in the mineral content and mainly in the organic content can make dentin more susceptible to demineralization [27].

In a previous study evaluating the cariogenic potential of different inoculum sources (dental biofilm and saliva), it was demonstrated that under identical *in vitro* conditions, the cariogenic potential becomes similar, regardless of the chosen source (saliva vs. biofilm, caries active vs. caries inactive donator) [22]. Considering that hyposalivation is one of the main consequences of head and neck radiotherapy, and that this condition can persist even 6 months after the end of treatment [28], we chose to collect dental biofilm in the present study.

Change in the oral microbiota of patients submitted to doses greater than 30 Gy has been detected [[28], [29], [30]], with a high level of *Lactobacillus* (58 %) and *mutans streptococci* (35 %) even 1 year after radiotherapy [29]. In the present study, there is a small difference in the results of CFU counting, with a slight increase of cariogenic bacteria in the irradiated biofilm, although these differences were not significant to influence the demineralization parameters, which may be due to the continuous sucrose exposure during microcosm biofilm formation. Thus, the irradiated biofilm in this experimental model appears to exert no influence on the formation of the initial dentin lesion. A recent work [31] demonstrated that the initial bacteriological profile of the inoculum did not interfere in the formation of the initial caries lesion under microcosm biofilm model, once the conditions for biofilm growth have been standardized *in vitro* [22,31]. If a less cariogenic model would have been applied, the distinction between the sources of microorganism could have been different. More specific techniques, such as biofilm microbiome, would be relevant for understanding eventual microbiological differences between dental biofilm sources (from irradiated vs. healthy patient, which means from patient with HNC vs. without cancer).

The experimental artificial saliva with proteins (Cane CPI-5 and hemoglobin) did not present promising results in the reduction of demineralization or not even in the reduction of the CFU counting when compared to DiW (negative control); thus, the secondary null hypothesis was accepted. In fact, the negative control group, with deionized water washing, showed better results than the tested experimental saliva. This fact could be explained by the washing effect of water, which could have disorganized the biofilm or/and remove the acid product, since this group was subjected to washing procedure twice (PBS and DiW). This also helps to justify differences in dentin demineralization between negative control vs. irradiated dentin/irradiated biofilm (not compared statistically), since irradiated dentin/irradiated biofilm group was not exposed to any type of washing.

Cane CPI-5 has shown promising results in preventing erosive wear (enamel and dentin) *in vitro* [32,33] and *in situ* at a concentration of 0.1 mg/ml [34]. Regarding the prevention of enamel caries, a recent study also under microcosm biofilm showed that different concentrations of Cane CPI-5 (0.05, 0.1 and 0.5 mg/ml) were effective in significantly reducing the integrated mineral loss when compared to the negative control (PBS) [12]. Furthermore, the lowest concentrations (0.05 and 0.1 mg/ml) were the most effective in reducing the CFU counting for cariogenic bacteria (*Lactobacillus* sp, total *streptococci* and *mutans streptococci*) [12]. When the Cane CPI-5 concentration was increased to 1 mg/ml, the protective antibacterial effect was lost [35]. The concentrations that seem to be most effective, both for preventing tooth erosion and for enamel caries control, are 0.05 and 0.1 mg/ml. However, we did not find similar results for dentin in our study, using a concentration of 0.1 mg/ml. Lower concentrations of Cane CPI-5 (0.025 and 0.05 mg/ml) have already been tested in microcosm biofilm for root dentin and they were not able to reduce the CFU counting for any bacterial group evaluated neither to reduce demineralization compared to negative control (PBS) [36].

Hemoglobin at a concentration of 1 mg/ml showed similar results to Cane CPI-5 in preventing erosive tooth wear *in vitro* [11]. In addition, the association of hemoglobin (1 mg/ml) and Cane CPI-5 (0.1 mg/ml) was the one that presented the best protective result against erosive tooth wear *in vitro* when compared to the isolated proteins [37]. Thus, the combination of both proteins could be more effective in preventing dental caries as well. No previous studies evaluated hemoglobin and the association between hemoglobin and Cane CPI-5 for preventing dental caries. However, our study did not show additive effect of the combination.

Differently from enamel, dentin has a higher organic content that, once exposed to demineralization, is subjected to the activation of matrix metalloproteinase (MMP) responsible for degrading its organic content. MMPs are considered important mediators of the degradation of the dentin organic matrix, being collagenases and gelatinases the most associated with dentin caries [38]. Radiotherapy has been associated with the increased expression and activation of MMPs in some tissues [38]. In addition, there is a greater presence of cariogenic bacteria in dentinal lesions compared to enamel lesions [39,40] as well as the presence of proteolytic bacteria [41], which could have digested the tested proteins added in experimental saliva and their derivates. All these factors may have influenced the low efficacy of the experimental saliva, and perhaps for the specific condition (dentin + radiotherapy) the concentration of Cane CPI-5 and hemoglobin should have been higher to those tested in the present study.

It is also important to consider that, despite the good results of proteins in previous studies with enamel, both Cane CPI-5 (pH 7.88) and hemoglobin were tested diluted in deionized water [11,12,34]. Only Pelá et al. [42] evaluated Cane CPI-5 incorporated into a chitosan gel (prepared with acetic acid), pH 5.5, against erosive tooth wear. When these proteins are added in a more complex vehicle in terms of the number of reagents, such as the artificial saliva used in the present work, many variables may influence their effect, such as the final pH of the product and possible interactions between components, both affecting the protein bioavailability. Accordingly, carboxymethylcellulose, which has a negative charge, could have interacted with the tested proteins, whose charge is positive [43]. Carboxymethylcellulose is added into artificial saliva since it plays an important role in improving oral lubrication [44]. More studies need to be carried out focusing on the maintenance of the protective action of proteins and their stability, minimizing possible interactions within dental products as gels, mousses, mouthwashes, and toothpastes. It was not possible to adjust the pH of the experimental saliva (pH values between 5.4 and 5.6) to be similar to the positive control – BioXtra (pH 6.3), since it could alter the stability and reactivity of the saliva.

The only saliva that had a protective potential was the positive control (BioXtra®), which has proteins lysozyme, lactoferrin and lactoperoxidase as active ingredients, with broad-spectrum bacteriostatic activity [45,46] and it is used to relieve symptoms of dry mouth after radiotherapy [44]. No study has evaluated saliva BioXtra® with respect to antimicrobial or anticaries effect. Formulations of toothpaste and gel (with the same active ingredients of artificial saliva) have shown to reduce the number of *Lactobacillus* sp. and *S. mutans* in previous clinical studies [46,47]. In addition, sodium monofluorophosphate (1500 ppm F^−^) is present in its composition, described as an inactive ingredient. Although described as an inactive ingredient, in our experimental model, fluoride into the artificial saliva might have reduced demineralization and increased remineralization [48]. The influence of the presence of fluoride in BioXtra® saliva should be studied in the future. As the work had focus on artificial saliva, we did not include F solution as a control group. This shall be done in the future to better understand the results.

## 5 Conclusion

In conclusion, the type of dentin (sound or irradiated) has impact on the development of dentin lesions using this study model, while the quality of the biofilm (from irradiated or non-irradiated patients) has no influence. Considering that no previous study had compared the impact of tooth irradiation and biofilm from irradiated patients on the dentin caries formation, our study highlighted the impact of the irradiation in increasing the susceptibility of dentin to demineralization.

None of the experimental saliva formulations containing proteins (hemoglobin and/or sugar cane cystatin) demonstrated antimicrobial and/or anticaries effects compared to deionized water (negative control). Other important finding is that BioXtra® saliva (positive control) is able to reduce dentin lesion development, while the experimental solutions were not, which should be confirmed in future studies.

## Funding

The authors B.M.S. and A.C.M. received scholarship and grant from The São Paulo Research Foundation (10.13039/501100001807FAPESP), respectively (grant number 2019/07241-0 and 2019/21797-0, respectively).

## Ethics approval

This study was approved by ethics committee on animal research of the Bauru School of Dentistry, University of São Paulo, Brazil (CEUA, Number: 004/2018). All procedures performed in studies involving human participants were in accordance with the ethical standards of the Institutional and/or National Research Committee (Ethics Committee of the Bauru School of Dentistry, University of São Paulo, Brazil, number 97497318.00000.5417) and with the 1964 Helsinki declaration and its later amendments or comparable ethical standards.

## Informed consent

Informed consent was obtained from all individual participants included in the study.

## Data availability statement

All data generated or analyzed during this study are included in this article. Further enquiries can be directed to the corresponding author.

## CRediT authorship contribution statement

**Beatriz Martines de Souza:** Writing – original draft, Visualization, Validation, Resources, Methodology, Investigation, Funding acquisition, Formal analysis, Data curation, Conceptualization. **Aline Silva Braga:** Writing – review & editing, Resources, Methodology, Investigation. **Mariele Vertuan:** Writing – review & editing, Validation, Resources, Investigation. **Susan Sassaki:** Validation, Resources, Investigation. **Tamara Teodoro Araújo:** Validation, Resources, Investigation. **Paulo Sergio da Silva Santos:** Writing – review & editing, Visualization, Validation, Methodology, Conceptualization. **Marilia Afonso Rabelo Buzalaf:** Writing – review & editing, Visualization, Validation, Methodology, Conceptualization. **Ana Carolina Magalhães:** Writing – review & editing, Writing – original draft, Visualization, Validation, Supervision, Resources, Project administration, Methodology, Funding acquisition, Conceptualization.

## Declaration of competing interest

We wish to confirm that there are no known conflicts of interest associated with this publication and there has been no significant financial support for this work that could have influenced its outcome.

We confirm that the manuscript has been read and approved by all named authors and that there are no other persons who satisfied the criteria for authorship but are not listed. We further confirm that the order of authors listed in the manuscript has been approved by all of us.

We confirm that we have given due consideration to the protection of intellectual property associated with this work and that there are no impediments to publication, including the timing of publication, with respect to intellectual property. In so doing we confirm that we have followed the regulations of our institutions concerning intellectual property.

We understand that the Corresponding Author is the sole contact for the Editorial process (including Editorial Manager and direct communications with the office). He/she is responsible for communicating with the other authors about progress, submissions of revisions and final approval of proofs. We confirm that we have provided a current, correct email address, which is accessible by the Corresponding Author.
