# Supplementary material for: Influence of irradiated dentin, biofilm and different artificial saliva formulations on root dentin demineralization
Source: Heliyon. 2024 Aug 14;10(16):e36334. doi: 10.1016/j.heliyon.2024.e36334 (PMC11378960; doi:10.1016/j.heliyon.2024.e36334)
Supplement: Multimedia component 1 [file mmc1.pdf]

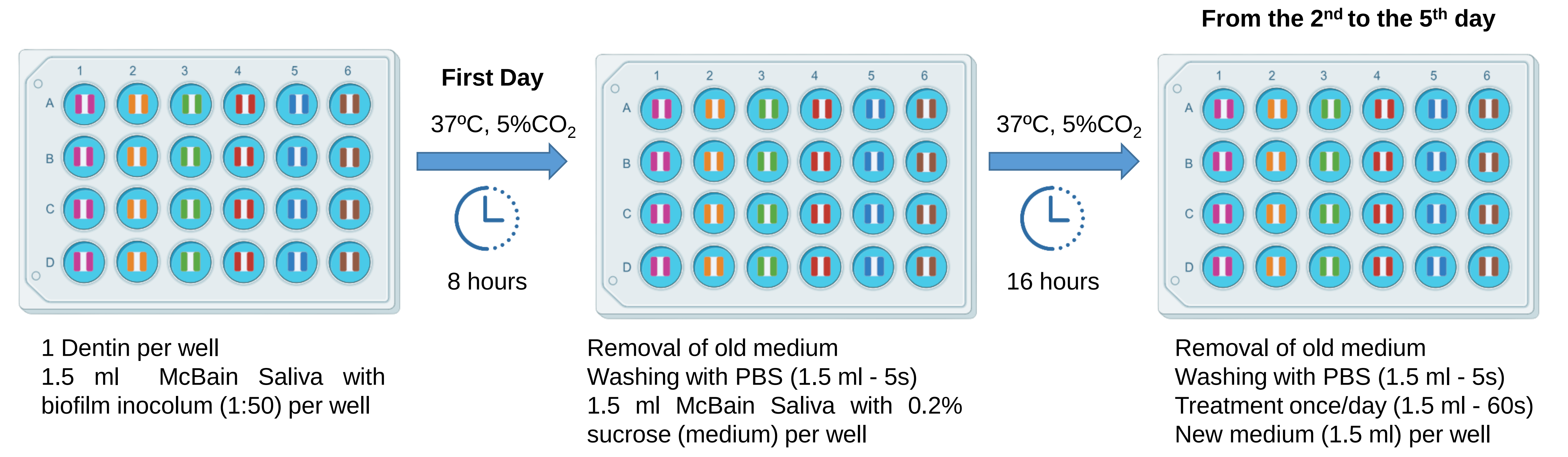

From the 2nd to the 5th day
First Day
37ºC, 5%CO_2
8 hours
37ºC, 5%CO_2
16 hours
1 Dentin per well
1.5 ml McBain Saliva with biofilm inocolum (1:50) per well
Removal of old medium
Washing with PBS (1.5 ml - 5s)
1.5 ml McBain Saliva with 0.2% sucrose (medium) per well
Removal of old medium
Washing with PBS (1.5 ml - 5s)
Treatment once/day (1.5 ml - 60s)
New medium (1.5 ml) per well
