# Supplementary material for: Influence of irradiated dentin, biofilm and different artificial saliva formulations on root dentin demineralization
Source: Heliyon. 2024 Aug 14;10(16):e36334. doi: 10.1016/j.heliyon.2024.e36334 (PMC11378960; doi:10.1016/j.heliyon.2024.e36334)
Supplement: Multimedia component 2 [file mmc2.pdf]

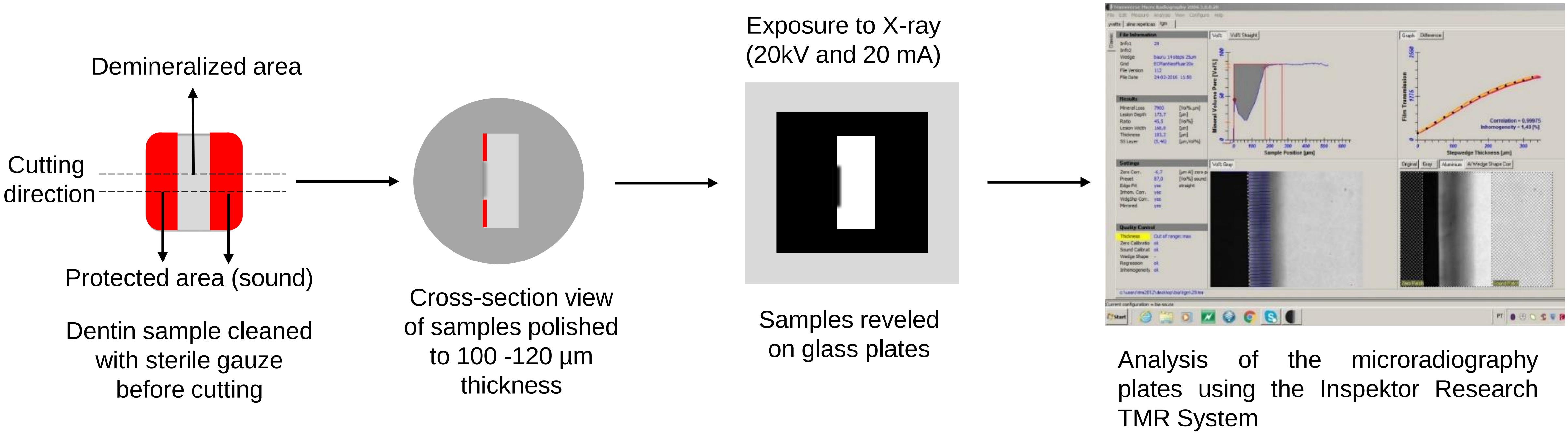

Demineralized area
Cutting direction
Protected area (sound)
Dentin sample cleaned with sterile gauze before cutting
Cross-section view of samples polished to 100 -120 µm thickness
Exposure to X-ray (20kV and 20 mA)
Samples reveled on glass plates
Analysis of the microradiography plates using the Inspektor Research TMR System
